# Stakeholder preferences for attributes of digital health technologies to consider in health service funding

**DOI:** 10.1017/S0266462323000089

**Published:** 2023-02-14

**Authors:** Amy von Huben, Martin Howell, Sarah Norris, Kam Cheong Wong, James Tang, Samia Kazi, Liliana Laranjo, Clara K. Chow, Kirsten Howard

**Affiliations:** 1 Sydney School of Public Health, The University of Sydney, Sydney, NSW, Australia; 2 Menzies Centre for Health Policy and Economics, The University of Sydney, Sydney, NSW, Australia; 3 School of Rural Health, The University of Sydney, Orange, NSW, Australia; 4 Bathurst Rural Clinical School, Western Sydney University, Bathurst, NSW, Australia; 5 Westmead Applied Research Centre (WARC), The University of Sydney, Westmead, NSW, Australia; 6 Department of Cardiology, Westmead Hospital, Sydney, NSW, Australia; 7 Community and Primary Health Care Practice and Digital Health, Western Sydney Primary Health Network, Blacktown, NSW, Australia

**Keywords:** digital health technology, remote monitoring, chronic disease self-management, best-worst scaling, health technology assessment

## Abstract

**Objectives:**

Health service providers are currently making decisions on the public funding of digital health technologies (DHTs) for managing chronic diseases with limited understanding of stakeholder preferences for DHT attributes. This study aims to understand the community, patient/carer, and health professionals’ preferences to help inform a prioritized list of evaluation criteria.

**Methods:**

An online best–worst scaling survey was conducted in Australia, New Zealand, Canada, and the United Kingdom to ascertain the relative importance of twenty-four DHT attributes among stakeholder groups using an efficient incomplete block design. The attributes were identified from a systematic review of DHT evaluation frameworks for consideration in a health technology assessment. Results were analyzed with multinomial models by stakeholder group and latent class.

**Results:**

A total of 1,251 participants completed the survey (576 general community members, 543 patients/carers, and 132 health professionals). Twelve attributes achieved a preference score above 50 percent in the stakeholder group model, predominantly related to safety but also covering technical features, effectiveness, ethics, and economics. Results from the latent class model supported this prioritization. Overall, connectedness with the patient’s healthcare team seemed the most important; with “*Helps health professionals respond quickly when changes in patient care are needed*” as the most highly prioritized of all attributes.

**Conclusions:**

It is proposed that these prioritized twelve attributes be considered in all evaluations of DHTs that manage chronic disease, supplemented with a limited number of attributes that reflect the specific perspective of funders, such as equity of access, cost, and system-level implementation considerations.

## Introduction

The COVID-19 pandemic has highlighted the potentially beneficial role of digital health technologies (DHTs) in helping patients with chronic disease self-manage their illness at home. The two most common technologies are (i) Telemonitoring at home and (ii) Web or mobile phone management programs. These technologies can differ widely in technical function, reliability, stability, connectedness with clinicians and health system data, safety, ease of use, access, clinical effectiveness, and the cost of implementation and usage. For health service providers in countries where a health technology assessment (HTA) approach is used to inform decisions on the public funding of new healthcare technologies, a question arises over the most important issues to consider when evaluating DHTs.

HTA models such as EUnetHTA’s HTA Core Model (1) provide checklists of questions to be specifically considered for non-DHTs (e.g., pharmaceuticals, diagnostics, and screening) over nine domains, from the current use of the technology to legal aspects. Our systematic review of DHT evaluation frameworks (2) identified recommendations for DHT-specific issues in all nine domains, particularly safety and ethical analysis. Recently, specific HTA frameworks for DHTs have been developed and used for funding decisions (e.g., UK’s ESF (2) and DTAC (3), Germany’s DiGA, and Finland’s Digi-HTA (4;5)), but none cover issues in all nine domains. In addition, to the best of our knowledge, there have been no studies over a broad cross-section of the general community, patients, carers, and health professionals, to understand the relative importance of the issues included in these DHT frameworks and recommended in the DHT evaluation literature. A vital enabler of the effectiveness of these technologies is stakeholder buy-in (6–8); hence, understanding stakeholder preferences is critical to the DHT evaluation process. The quantitative discrete choice analysis methodology employed in this study allows for estimating the relative preference of many issues over a large sample of stakeholders.

This best–worst survey aims to understand stakeholder preferences for attributes of the technology relevant to the public funding of DHTs by health service providers. Identifying areas of agreement on the priority of the issues to be considered, and any areas of divergence by specific population groups, will enable us to develop a prioritized and specific DHT checklist to accompany standard HTA evaluation checklists and assist publicly funded health service providers in their evaluations.

## Methods

### Study population

Adult survey respondents were recruited from Australia, New Zealand, Canada, and the United Kingdom, given they have government-funded healthcare systems and use an HTA approach to inform public funding decisions. Respondents were recruited by an online research panel administered by an external organization (Dynata LLC, Shelton, CT, USA). Quota sampling by country and the absence or presence of chronic disease was used to obtain a representative sample of chronic disease patients and the general community in these four countries. Respondents could indicate they were all/any of chronic disease patients, carers of chronic disease patients, and health professionals, or if none of these, a general community member. Health professionals were recruited by advertising in Australian and international health professional network newsletters and member email lists. Networks covered general practice, specialists, nurses and nurse practitioners, health service researchers, and guidelines international network (GIN) members. Links to the survey website were provided for online survey completion in English. The study was approved by the University of Sydney Human Research Ethics Committee (Project Number: 2021/847). Data were collected from January to April 2022.

### Best–worst scaling

Case 1 “object” best–worst scaling (BWS) is a type of discrete choice experiment that allows for the measurement of relative preferences for various attributes on the same scale, providing better discrimination than the use of rating scales (9;10). In our survey, respondents repeatedly chose two objects in varying sets of three (where the “objects” are the DHT attributes) that represent the most perceptual difference, for example, the “most” and “least important,” on the continuum of attributes (10). The probabilities of the respondents’ choices were modeled using the multinomial logit (MNL) model, where the model coefficients (β) can be interpreted as the relative preferences for the attributes (10). Robust standard errors were used in estimation to allow for the correlation from individuals completing more than one choice set (11).

### Development of the issues and DHT attributes

To identify issues to be considered when evaluating DHTs for funding, we conducted an extensive systematic review of international peer-reviewed and gray literature to identify evaluation frameworks specific to DHTs that manage chronic disease at home (12). We compiled comprehensive lists of the most frequently recommended content across a nine-domain HTA and refined this into a more practical set of questions for each issue by applying them to a systematic review of recent primary research studies (13).

Because the list of issues and associated questions can be repetitive over HTA domains, they were grouped and translated into a set of non-overlapping DHT attributes that most represented the grouped issues. Several iterations were undertaken to form these attributes, with A.v.H. drafting, M.H., S.N., and C.C. reviewing, and K.H. resolving conflicts. A pilot of the BWS was also undertaken with a mix of fifteen patients, carers, health professionals, and general community members, who commented on whether they thought the attributes represented multiple issues, duplicated issues, or omitted essential issues. In response, attributes were modified. See Supplementary Table 1 for the final list of twenty-four DHT attributes. References to the DHT evaluation framework literature and the EUnetHTA Core Model domains and issue identifiers (1) that suggested the content are given for each attribute/grouped issue for traceability with our prior work (12;13) and integration with the EUnetHTA model.

### Survey design

SAS V9.4 (SAS Institute Inc., Cary, NC, USA) was used to find a statistically D-efficient nearly balanced incomplete block design, minimum sample size *n* = 288, resulting in ninety-six choice sets with three objects, blocked into eight versions of twelve questions to minimize survey fatigue and improve response efficiency (14). The surveys were programmed into Qualtrics (Qualtrics Software, Provo, UT, USA). Minor changes to the survey questions to minimize respondent completion time were made after piloting. The final survey was structured as follows: Participant information, consent, socio-demographics, patient/health professional experience with DHTs, explanation of the choice task with definitions of key terms, the scenario, and an example of a completed choice task (Supplementary Figure 1). Participants completed twelve choice questions and were asked to list any other important issues not included in the choice task. The survey concluded with questions on access to the internet, the level of help required when using personal digital technology, and one eHealth literacy question (15).

### Analysis

Participant characteristics were summarized. The number of participants who responded to “list any other important issues not included in the choice task” was counted, and responses were examined.

The sequential best–worst (16) multinomial logit (SBWMNL) model and panel latent class SBWMNL were estimated in R Statistical Software (v4.1.2; R Core Team 2021) (17) using the Apollo R package (v0.2.4) (11;18). In sequential best–worst, the participant is assumed in each choice question to pick the “best” choice first and then the “worst” out of the remaining choices. A reverse of this assumption was also tested with little change in the relative preferences.

Because we were interested in the extent of consensus and divergence in preferences over these stakeholder groups, models were constructed and tested for model fit by adding interaction terms to capture stakeholder groups in various combinations, that is, two stakeholder groups ((i) health professionals and (ii) all others), three groups ((i) health professionals, (ii) patients and carers, and (iii) community members), four groups (with the fourth group being respondents that were carers but not patients, previously in group 2). Health professionals who are also carers or patients will have additional knowledge and experiences of the healthcare system compared to non-health professionals. To reduce any potential risk of bias from this additional knowledge and experience in the patient and carer groups, these small number of health professionals were classified into the health professional group. Model fit was assessed by Likelihood-Ratio Tests and Akaike information criterion (AIC). The regression coefficients of the MNL model provide the relative importance of each attribute on the same scale and were scaled to 0–100 (least to most important) for ease of interpretation. The scaled coefficients are denoted as “preference scores” in tables and figures. To identify the most important issues for inclusion in a practical HTA checklist, we set an arbitrary preference score cut-off of fifty (50 percent) in at least one stakeholder group to determine the “prioritized” attributes.

A panel latent class SBWMNL model is a common technique used in choice modeling to investigate the heterogeneity of preferences that may not be captured in the base SBWMNL model. In this model, additional classes were added until the model with the best fit was found, with model fit assessed by the Bayesian Information Criterion (BIC) and the proportion of participants classified in each latent class with a posterior probability above 75 percent; the higher the proportions at these levels the lower the number of participants not ambiguously classified in each latent class (19;20). Although participants belong in a class to a certain probability in a latent class model, the model allows for examining associations between covariates (e.g., age and gender) and class membership probability. To identify these associations, all variables collected on all participants (i.e., not patient, carer, and health professional-specific) were entered into the class membership model, and variables with the highest *p*-value were removed sequentially until the best model fit was found based on the AIC. Before fitting the model, covariates with low numbers of respondents in specific categories were grouped with the most relevant neighboring response category for model stability; that is, the twenty-three students in employment status were grouped with part-time and casual workers, the eleven participants residing in “Other” countries were grouped with the United Kingdom, given they were residing in European countries, and responses to how often help was required when using technology were collapsed into never/rarely and sometimes/often/always. R packages Gmisc for plot and table output (21) and knitr (22) for reproducible research were also used in the analysis.

## Results

### Participants

A total of 1,317 participants consented, 27 completed fewer than three choice questions, and 39 surveys were excluded due to survey completion occurring in one-third of the median time (identified as “speeders” or possible bots), leaving 1,251 (95 percent) surveys for analysis. Respondent characteristics are reported in Table 1. Respondent types were 576 (46 percent) community members, 543 (43 percent) patient/carers (397 patients and 146 carers), and 132 (11 percent) health professionals. Participants were aged 18 to 80 plus years and 54 percent of the sample was female. While community members and patients/carers were evenly spread across the four countries, nearly half the health professionals were Australian, reflecting the health professional networks available to us. Twenty-one percent of participants lived in rural or remote areas. Although over 90 percent of participants spoke mainly English and 18 percent spoke a second language at home.

**Table 1.** Demographics of survey respondents by stakeholder group (*N* = 1,251)

	Community member *N* = 576	Patient/carer *N* = 543	Health professional *N* = 132
Age group			
18–39	143 (24.8%)	134 (24.7%)	58 (43.9%)
40–69	298 (51.7%)	301 (55.4%)	72 (54.5%)
70 and over	135 (23.4%)	108 (19.9%)	2 (1.5%)
Gender			
Male	285 (49.8%)	243 (44.8%)	38 (29.0%)
Female	287 (50.2%)	299 (55.2%)	93 (71.0%)
Country of residence			
Australia	145 (25.2%)	129 (23.8%)	64 (48.5%)
Canada	150 (26.0%)	138 (25.4%)	15 (11.4%)
New Zealand	143 (24.8%)	124 (22.8%)	23 (17.4%)
United Kingdom	135 (23.4%)	150 (27.6%)	23 (17.4%)
Other	3 (.5%)	2 (.4%)	7 (5.3%)
Location			
Rural or remote	127 (22.0%)	119 (21.9%)	19 (14.4%)
Urban	449 (78.0%)	424 (78.1%)	113 (85.6%)
Main language spoken at home			
English	529/576 (91.8%)	514/543 (94.7%)	112/130 (86.2%)
Speak a second language at home			
Yes	92/576 (16.0%)	87/543 (16.0%)	43/130 (33.1%)
Employment status			
Full time	223/576 (38.7%)	212/543 (39.0%)	84/130 (64.6%)
Part time/casual	70/576 (12.2%)	63/543 (11.6%)	40/130 (30.8%)
Not employed/unable to work	68/576 (11.8%)	95/543 (17.5%)	2/130 (1.5%)
Retired	204/576 (35.4%)	165/543 (30.4%)	0/130 (0.0%)
Student	11/576 (1.9%)	8/543 (1.5%)	4/130 (3.1%)
Highest level of education			
Primary school	9/576 (1.6%)	6/543 (1.1%)	0/130 (0.0%)
Secondary/high school	208/576 (36.1%)	162/543 (29.8%)	9/130 (6.9%)
Professional certificate	131/576 (22.7%)	141/543 (26.0%)	16/130 (12.3%)
Undergraduate/bachelor’s degree	155/576 (26.9%)	170/543 (31.3%)	39/130 (30.0%)
Postgraduate degree (master/doctoral)	73/576 (12.7%)	64/543 (11.8%)	66/130 (50.8%)
Internet access			
Have mobile internet access	473/576 (82.1%)	453/543 (83.4%)	121/130 (93.1%)
Have home internet access	545/576 (94.6%)	511/543 (94.1%)	123/130 (94.6%)
On a typical day, for how many hours do you have internet access?			
Median (IQR)	24.0 (8.0–24.0)	24.0 (10.0–24.0)	24.0 (20.2–24.0)
Missing	0 (0%)	0 (0%)	2 (1.5%)
How often do you need someone to help you when using your computer, mobile phone, tablet, or smart watch?			
Never	265/575 (46.1%)	225/542 (41.5%)	49/130 (37.7%)
Rarely	193/575 (33.6%)	190/542 (35.1%)	49/130 (37.7%)
Sometimes	79/575 (13.7%)	88/542 (16.2%)	19/130 (14.6%)
Often	21/575 (3.7%)	23/542 (4.2%)	7/130 (5.4%)
Always	17/575 (3.0%)	16/542 (3.0%)	6/130 (4.6%)
How often do you use the internet to find health information?			
Not at all	63/575 (11.0%)	33/543 (6.1%)	3/130 (2.3%)
A few times a year	296/575 (51.5%)	209/543 (38.5%)	13/130 (10.0%)
A few times a month	145/575 (25.2%)	180/543 (33.1%)	26/130 (20.0%)
A few times a week	53/575 (9.2%)	84/543 (15.5%)	35/130 (26.9%)
Daily	18/575 (3.1%)	37/543 (6.8%)	53/130 (40.8%)
Patient and carer survey			
Patient		397/543 (73.1%)	
Carer		146/543 (26.9%)	
Patient (or patient cared for) chronic conditions			
Cardiovascular/heart conditions		134/543 (24.7%)	
Diabetes		170/543 (31.3%)	
Digestive system conditions (e.g., Crohn’s, Celiac disease)		70/543 (12.9%)	
Nervous system conditions (e.g., Parkinson’s disease)		53/543 (9.8%)	
Chronic pain of unknown cause		89/543 (16.4%)	
Cancer		56/543 (10.3%)	
Kidney disease		33/543 (6.1%)	
Musculoskeletal system conditions (e.g., arthritis, back problems)		165/543 (30.4%)	
Obesity		95/543 (17.5%)	
Respiratory conditions (e.g., Chronic obstructive pulmonary disease (COPD), asthma)		146/543 (26.9%)	
Other/s chronic disease		37/543 (6.8%)	
Patient (or patient cared for) use of DHT			
Currently use a digital health technology to manage my/their chronic disease		117/543 (21.5%)	
Do not use one currently, but want to use a digital health technology		167/543 (30.8%)	
Have used a digital health technology in the past, but not currently		36/543 (6.6%)	
None of the above		223/543 (41.1%)	
Health professional survey			
I manage/treat patients with chronic disease			86/132 (65.2%)
I have purchased digital health technologies for my health service to manage/treat patients with chronic disease			22/132 (16.7%)
I am looking to purchase digital health technologies for my health service to manage/treat patients with chronic disease			19/132 (14.4%)
None of above			31/132 (23.5%)

*Note*: Non-binary: community member 4 (.7%), patient or carer 1 (.2%); prefer not to say: health professional 1 (.8%).

In terms of internet access (Table 1), over 80/95 percent had mobile/home internet access. However, areas of inequity in access were observed, with a quarter of community members and patients/carers having ten or fewer hours of available internet access per day. Regarding perceived competency with personal-use digital technologies, only 7 percent stated they often or always needed someone to help them when using these technologies. Concerning e-Health literacy, a substantially higher proportion of health professionals reported using the internet daily to find health information than other groups.

For the patient and carer survey (Table 1), there was a broad representation of common chronic diseases. Twenty-two percent of patients, or the person they cared for, were currently using a DHT to manage their condition, nearly one-third wanted to use a DHT but did not currently, and only 7 percent had used one in the past but not currently. For the health professional survey (Table 1), nearly two-thirds managed or treated patients with chronic disease, 17 percent had purchased a DHT for their health service to manage or treat patients with chronic disease, and 14 percent were looking to purchase such a DHT for their health service.

Thirty-one percent (394) of participants responded to “list any other important issues not included in the choice task.” Noting that the choice task was designed so participants would not see all attributes, most issues raised were already included issues (e.g., easy to use) or sub-issues (e.g., consider user age). Only one issue outside of those included, cultural safety, was raised by more than one participant (three health professionals).

### Preferences for attributes

**Figure 1.**
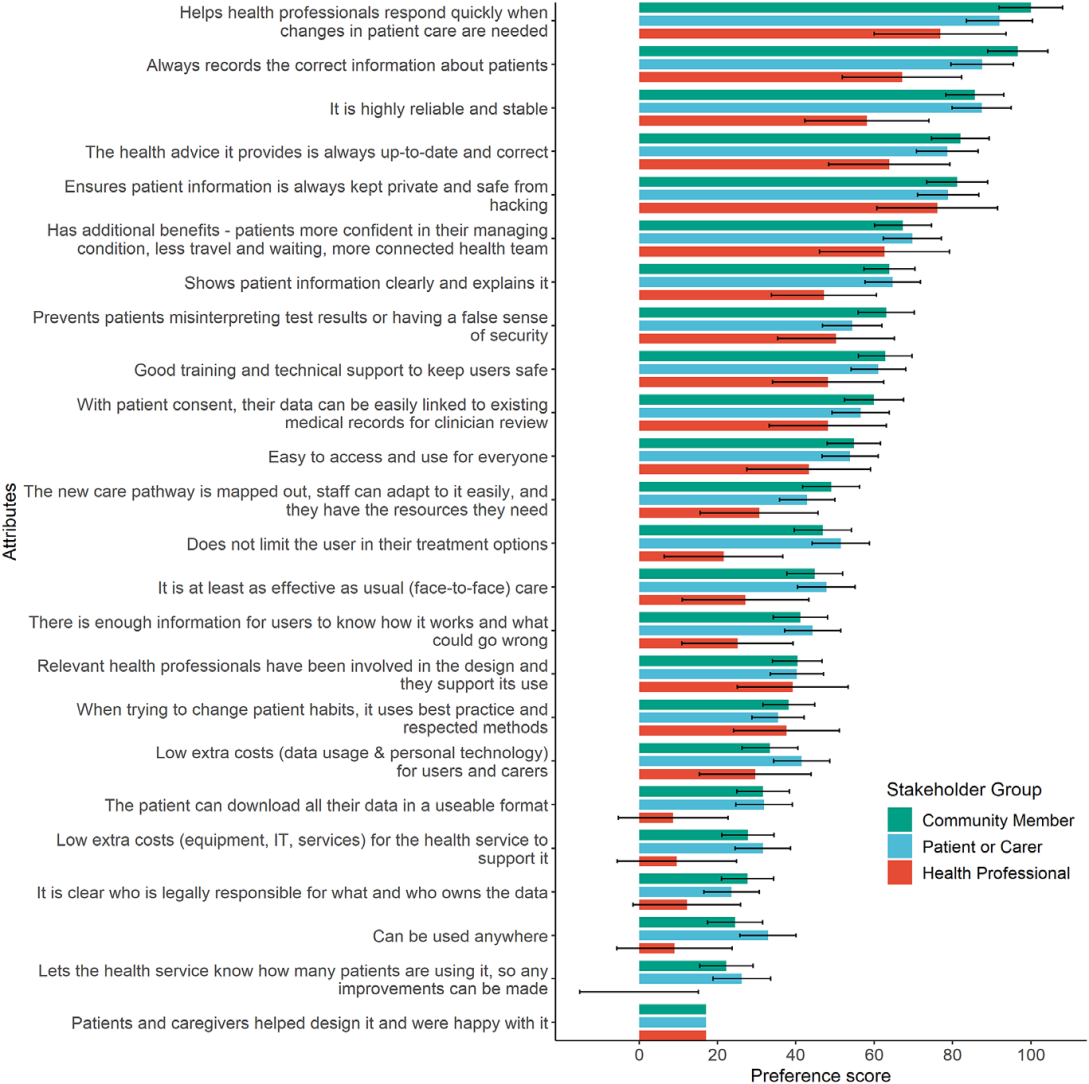
Mean preference scores and 95% confidence intervals for DHT attributes from sequential best–worst multinomial model with three stakeholder groups. Preference scores are coefficients scaled from 0 to 100 (least important to most important).

For the SBWMNL model, the best fit based on the Likelihood-Ratio Test and AIC included interactions for three stakeholder groups; indicating sufficient preference differences between community members, patient/carers, and health professionals, but insufficient difference between patients and carers to warrant a separate carer group. Model results with scaled preference scores (0 to 100; least to most important) are displayed in Table 2. Mean and 95% confidence interval preference scores are plotted by stakeholder group in Figure 1. Even though statistical differences exist, the preferences indicate a similar ranking between community members and patients/carers. While health professionals had some differing priorities, all stakeholder groups agreed on the eleven most important DHT attributes, six being in the Safety HTA domain. The most important attribute for all stakeholder groups was “*It helps health professionals respond quickly when changes in patient care are needed”* (Table 2).

**Table 2.** Relative preferences of attributes from sequential best–worst multinomial model with three stakeholder groups: community member, patient/carer, and health professional

		Community member (*N* = 576)				Patient/carer (*N* = 543)				Health professional (*N* = 132)			
HTA domain^a^	Attributes	*β*	(95% CI)	Preference score	*p*	*β*	(95% CI)	Preference score	*p*	*β*	(95% CI)	Preference score	*p*
SAF	Helps health professionals respond quickly when changes in patient care are needed	2.03	(1.84, 2.23)	100.0	<.001	1.84	(1.63, 2.04)	92.0	<.001	1.47	(1.05, 1.88)	76.9	<.001
TEC/SAF	Always records the correct information about patients	1.95	(1.76, 2.14)	96.7	<.001	1.73	(1.53, 1.93)	87.6	<.001	1.23	(.85, 1.60)	67.1	<.001
SAF	It is highly reliable and stable	1.68	(1.50, 1.86)	85.7	<.001	1.73	(1.54, 1.91)	87.5	<.001	1.01	(.62, 1.40)	58.1	<.001
EFF	The health advice it provides is always up-to-date and correct	1.59	(1.41, 1.77)	82.0	<.001	1.51	(1.32, 1.70)	78.7	<.001	1.15	(.77, 1.53)	63.9	<.001
SAF/ETH	Ensures patient information is always kept private and safe from hacking	1.57	(1.38, 1.76)	81.2	<.001	1.52	(1.32, 1.71)	78.9	<.001	1.45	(1.07, 1.83)	76.1	<.001
EFF/ECO	Has additional benefits – patients more confident in their managing condition, less travel and waiting, more connected health team	1.23	(1.05, 1.41)	67.3	<.001	1.29	(1.11, 1.47)	69.8	<.001	1.12	(.71, 1.52)	62.6	<.001
TEC	Shows patient information clearly and explains it	1.15	(.99, 1.31)	63.9	<.001	1.17	(1.00, 1.34)	64.7	<.001	.74	(.41, 1.07)	47.2	<.001
ETH	Prevents patients misinterpreting test results or having a false sense of security	1.13	(.95, 1.30)	63.1	<.001	.91	(.73, 1.10)	54.4	<.001	.81	(.45, 1.18)	50.3	<.001
TEC	Good training and technical support to keep users safe	1.12	(.96, 1.29)	62.9	<.001	1.08	(.91, 1.25)	61.1	<.001	.76	(.42, 1.11)	48.2	<.001
TEC/SAF	With patient consent, their data can be easily linked to existing medical records for clinician review	1.05	(.87, 1.24)	60.0	<.001	.97	(.79, 1.15)	56.6	<.001	.76	(.40, 1.13)	48.2	<.001
CUR/SAF/EFF/ETH	Easy to access and use for everyone	.93	(.76, 1.09)	54.9	<.001	.90	(.73, 1.08)	53.9	<.001	.64	(.26, 1.03)	43.3	.001
ORG	The new care pathway is mapped out, staff can adapt to it easily, and they have the resources they need	.78	(.61, .96)	49.0	<.001	.63	(.46, .81)	42.9	<.001	.33	(−.04, .70)	30.6	.078
ETH	Does not limit the user in their treatment options	.73	(.55, .91)	46.9	<.001	.84	(.66, 1.02)	51.5	<.001	.11	(−.26, .48)	21.6	.564
EFF	It is at least as effective as usual (face-to-face) care	.68	(.51, .86)	44.8	<.001	.75	(.57, .93)	47.8	<.001	.25	(−.15, .64)	27.2	.223
SAF	There is enough information for users to know how it works and what could go wrong	.59	(.42, .76)	41.2	<.001	.67	(.49, .84)	44.3	<.001	.20	(−.15, .55)	25.1	.270
ORG	Relevant health professionals have been involved in the design and they support its use	.57	(.42, .73)	40.4	<.001	.57	(.40, .74)	40.3	<.001	.54	(.20, .89)	39.2	.002
EFF	When trying to change patient habits, it uses best practice and respected methods	.52	(.36, .68)	38.2	<.001	.45	(.29, .61)	35.5	<.001	.50	(.17, .83)	37.6	.003
CUR	Low extra costs (data usage and personal technology) for users and carers	.40	(.23, .58)	33.4	<.001	.60	(.42, .77)	41.5	<.001	.31	(−.04, .66)	29.7	.084
TEC	The patient can download all their data in a useable format	.36	(.19, .52)	31.7	<.001	.36	(.19, .54)	31.9	<.001	−.21	(−.55, .14)	8.7	.240
TEC/ECO	Low extra costs (equipment, IT, services) for the health service to support it	.26	(.10, .43)	27.8	.002	.36	(.18, .53)	31.6	<.001	−.18	(−.56, .19)	9.6	.335
LEG	It is clear who is legally responsible for what and who owns the data	.26	(.10, .42)	27.7	.002	.16	(−.01, .33)	23.6	.073	−.12	(−.46, .22)	12.2	.483
EFF/ETH/SOC	Can be used anywhere	.18	(.01, .35)	24.5	.039	.39	(.21, .56)	32.9	<.001	−.20	(−.56, .16)	9.0	.283
CUR	Let’s the health service know how many patients are using it, so any improvements can be made	.13	(−.04, .29)	22.3	.137	.22	(.04, .40)	26.2	.015	−.42	(−.79, −.05)	0.0	.027
EFF	Patients and caregivers helped design it and were happy with it	Reference				Reference				Reference			

*Note*: Model fit: Likelihood-Ratio Test (LRT) *p*-value = .003 (three stakeholder groups versus two stakeholder groups model; where two stakeholder groups versus constant only model LRT *p*-value <.001, and four stakeholder groups versus three stakeholder groups model LRT *p*-value = .99), Pseudo *r* ^2^ .074, Akaike information criterion (AIC) 49322.09 (lowest out of constant only model: 49341.94, two groups model: 49322.42, four groups model: 49369.64), Bayesian information criterion (BIC) = 49895.46 (second lowest out of constant only model: 49533.06, two groups model: 49704.67, four groups model: 50134.14), Respondents *n* = 1,251 (576, 543, and 132 observations), *β =* regression model coefficient estimates.

a HTA domain, health technology assessment (HTA) domains of the EUnetHTA HTA Core Model version 3.0 (1):

CUR Describes the new technology’s target population, target condition and current management, current and expected utilization, and regulatory status.

ECO Provides information on the new technology’s costs, health-related outcomes, and economic efficiency to inform value for money judgments.

EFF Provides evidence of comparative effectiveness of the new technology in producing health benefits in the relevant healthcare setting.

ETH Considers potential harms to autonomy, respect for persons, justice, and equity from the use of the new technology or from performing the HTA.

LEG Identifies rules and regulations protecting patient’s rights and societal interests for consideration when evaluating the new technology.

ORG Identifies resources to mobilized or organized to implement the new technology and the consequences (intra-/inter-organizational and health system).

SAF Identifies unwanted or harmful effects of the new technology important to patients or the decisions of healthcare providers and policymakers.

SOC Considers issues related to the new technology relevant to patients, carers, and social groups.

TEC Describes the new technology’s features in enough detail to differentiate it from comparators, and the investments, tools, and training required to use it.

Using our criteria for prioritized attributes, twelve attributes achieved a preference score above fifty in at least one stakeholder group (Table 3). From Figure 1, we can see the only statistically significant differences in these twelve prioritized attributes are where health professionals have lower preference scores for the attributes “*It is highly reliable and stable*” and “*Does not limit the user in their treatment options*” than patients/carers and community members.

**Table 3.** Prioritized attributes for consideration in an evaluation of a digital health technology (DHT)

#	Attribute	HTA domain	Highest preference score
1	Helps health professionals respond quickly when changes in patient care are needed	SAF	100.0
2	Always records the correct information about patients	TEC/SAF	96.7
3	Is highly reliable and stable	SAF	87.5
4	The health advice it provides is always up-to-date and correct	EFF	82.0
5	Ensures patient information is always kept private and safe from hacking	SAF/ETH	81.2
6	Has additional benefits – patients more confident in their managing condition, less travel and waiting, more connected health team	EFF/ECO	69.8
7	Shows patient information clearly and explains it	TEC	64.7
8	Prevents patients misinterpreting test results or having false sense of security	ETH	63.1
9	Good training and technical support to keep users safe	TEC	62.9
10	With patient consent, their data can be easily linked to existing medical records for clinician review	TEC/SAF	60.0
11	Easy to access and use for everyone	CUR/SAF/EFF/ETH	54.9
12	Does not limit the user in their treatment options	ETH	51.5

Outside of the twelve prioritized attributes, there were more differences in relative priorities between stakeholder groups. Relative to other stakeholder groups, health professionals had a higher preference for “*Relevant health professionals involved in design and support its use*” (organizational aspects) and “*When trying to change patient habits, it uses best practice and respected methods*” (clinical effectiveness), whereas community members had a higher preference for “*The new care pathway is mapped out, staff can adapt to it easily, and they have the resources they need*” (organizational aspects), and patients/carers had a higher preference for “*it is at least as effective as usual (face-to-face) care*” (clinical effectiveness).

The least preferred attributes of all stakeholder groups were reporting actual use of the health service to prompt improvements (current use), having patients and caregivers involved in the design and satisfied with the DHT (clinical effectiveness), being able to use the DHT anywhere (clinical effectiveness, ethical analysis, and social aspects), clarity on who is legally responsible and who owns the data (legal aspects), low additional costs for the health system to support it and patients and carers to use it (current use and costs), and the ability for the patient to download and use their data (technical characteristics).

### Latent class analysis

Latent class SBWMNL models with between one to four classes were examined; the model with three classes provided the lowest BIC, with at least 62 percent of participants classified in each latent class with a posterior class-membership probability above 75 percent. The covariates predictive of class membership were stakeholder group, gender, age, country of residence, speaking a second language at home, employment status, and how often a participant needed to ask for someone’s help using personal digital technologies. Rural versus urban location, highest level of education, access to home or mobile internet, and the frequency of internet use to find health information were not predictive of class membership. Model results for the final model are displayed in Supplementary Table 2 and Supplementary Figure 2.

Class 1 had similar preference scores across all attributes, showing no clear preferences. Class 2 was characterized by their top concern being the privacy of patient information and was the only group to show a preference for considering legal responsibility and who owns the data. Class 3 most preferred the ability of the DHT to “*help health professionals to respond quickly when changes to patient care are needed*” and preferred a similar top eleven attributes as in the base case MNL model, except for preferring “*It is at least as effective as usual (face-to-face) care*” over patient information privacy and ease of access and use.

Statistically significant differences in socio-demographics between classes existed by gender, age group, how often you needed someone’s help when using personal digital technologies, and employment status (Supplementary Table 2). Moderate evidence of differences existed by stakeholder group, speaking a second language at home, and country of residence. Class 2 was less likely to be patients or carers than Class 3, OR .63 (95% CI .41, .98). Class 1 was less likely to be female, OR .53 (.33, .85), and less likely to be in the older age categories (40–69 years old OR .44 (.25, .75); 70 years or older OR .16 (.05, .46)) than Class 3. Likely related to their younger age, Class 1 was also less likely to be in part-time/casual employment or retired. Class 1 was also more likely to state they needed someone’s help more frequently to use their personal digital technologies OR 5.30 (2.98, 9.42) than Class 3 and was more likely to speak a second language at home.

## Discussion

Our results indicate a broad level of agreement amongst stakeholder groups and little heterogeneity from the latent classes regarding the twelve most important attributes (preference score above fifty in at least one stakeholder group) for health services to consider when funding DHTs for patients with chronic disease to use at home (Table 3). Six of these attributes were in the safety HTA domain. Above all, the theme of connectedness with a patient’s healthcare team seems the most important with “*Helps health professionals respond quickly when changes in patient care are needed*” the most preferred of all attributes, along with *“Has additional benefits – patients more confident in their managing condition, less travel and waiting, more connected health team”* and “*With patient consent, their data can be easily linked to existing medical records for clinician review*” having preference scores above sixty. Our findings are important because attributes such as “*Helps health professionals respond quickly when changes in patient care are needed*” and “*Does not limit the user in their treatment options*” are not a focus of existing HTA frameworks for DHTs. Outside of the top twelve, some attributes were preferred by only one stakeholder group, which may be important to consider if prioritizing issues for a particular stakeholder. Relative to other stakeholder groups, community members preferred the health care pathway being mapped out and well-resourced; patients/carers preferred the DHT being at least as effective as face-to-face care, and health professionals preferred relevant health professionals being involved in designing and supporting the use of the DHT, and that behavior change techniques used by the DHT should represent best practice.

The strength of the survey was the large sample and broad cross-section of stakeholders. Nonetheless, there are limitations to be noted. The smaller sample size for health professionals resulted in larger confidence intervals which lowered our ability to prioritize the least important attributes. Our sample countries may limit the generalizability of our results to some countries, such as low- and middle-income countries, but are likely to be generalizable to countries where a HTA approach is used to inform public funding decisions on health care. How we framed the scenario with the DHTs approved by the government for efficacy and safety (Supplementary Figure 1) may have made participants less concerned about safety. However, six of the twelve prioritized attributes were from the safety HTA domain. Stating that the DHTs had the same price may have made participants not consider additional cost attributes as important, but this was necessary for participants to consider what attributes maximized value for the same price. Stating that studies had found the DHTs to be equally effective may also have made participants deprioritize being equally effective as usual (face-to-face) care. However, this attribute was still rated quite highly in latent class groups. The phrasing of the attributes may have biased responses or misrepresented the issues; however, piloting the survey with feedback and subsequent changes and the large sample sizes should have lessened these risks.

Our objective was to identify the most important attributes to stakeholders to make it practical to consider all the underlying issues for these prioritized attributes, as listed in Supplementary Table 1, when evaluating a DHT for public funding. Not all issues in this list will always be relevant, but they should be considered. We aimed to develop a practical and focused list of DHT-specific considerations over the nine domains of the EUnetHTA model. We found evidence that this list should prioritize safety domain DHT issues, especially connectedness with clinician and health care records with appropriate privacy controls, along with clinical effectiveness domain issues such as keeping health advice up to date and correct, and the additional benefits that DHTs can bring for patient self-confidence, less travel and waiting, and a more connected health team. Technical features, such as displaying and explaining patient information clearly, and ethical issues, such as preventing patients from misunderstanding test results or having a false sense of security, were also highly rated.

In prioritizing these issues, we may omit consideration of other issues recommended when evaluating DHTs (12), and it must be noted that our cut-off for prioritized attributes at 50 percent is somewhat arbitrary. However, preference score results for all attributes are shown so users can set their own cut-off if needed. Some of these lower priority issues most affect the health services directly, such as additional costs to support DHTs, legal responsibilities, ownership of data, and monitoring patient usage to prompt improvement or replacement of ineffective DHTs. If health service providers had been included as respondents, we may have observed higher preferences for these attributes. However, the survey context was to specifically identify community, patient/carer, and health professional preferences.

As to issues that directly impact the surveyed stakeholders, such as having enough information for users to know how it works and what could go wrong, and patients being able to download and use their own data, we conclude these are not as important and have not been included in the focused list. Surprisingly, being able to use the DHT anywhere, which may affect people in areas of low connectivity, was given a low preference across all groups with no evidence of a higher preference from rural/remote participants. This attribute may be seen as being captured by “*Easy to access and use for everyone*,” which rated higher in all groups.

Given the mounting evidence of the benefits of consumer involvement in health research (23–25), in recent years, we have seen the establishment of consumer councils in healthcare organizations, HTA consumer consultative committees, and research funding agencies requiring evidence of consumer involvement. Therefore, it was unexpected that the attribute “*Patients and caregivers helped design it and were happy with it*” was rated so low by community members and patients/carers. This result may reflect the late and slow cultural shift toward involving consumers in health care compared to other areas of the economy, particularly digital health (26;27). Our results should not be taken to suggest that consumer involvement in codesign and HTA is unimportant. On the contrary, our results show that consumers prioritize other attributes over having their say or their needs met. They bring different perspectives on priorities for digital health attributes, enhancing the design and HTA process.

## Conclusion

We observed broad consensus among community members, patients/carers, and health professionals on the most important attributes to be considered by health service providers when funding DHTs for patients with chronic disease to use at home. Twelve primary attributes, mainly in the safety HTA domain and with a priority for connectedness with a patient’s healthcare team, were identified as most important by the stakeholder groups. As existing HTA frameworks for DHT currently do not cover all these prioritized issues, we aim to develop a practical list of DHT-specific considerations over the nine domains of the EUnetHTA model.

## Supplementary material

For supplementary material accompanying this paper visit https://doi.org/10.1017/S0266462323000089. click here to view supplementary material
