# Supplementary material for: Stakeholder preferences for attributes of digital health technologies to consider in health service funding
Source: Int J Technol Assess Health Care. 2023 Feb 14;39(1):e12. doi: 10.1017/S0266462323000089 (PMC11574528; doi:10.1017/S0266462323000089)
Supplement: Supplementary file 1 [file S0266462323000089sup001.docx]

Supplementary Table 1: Mapping of DHT attributes to DHT issues

| # | **Attribute** | **Grouped issue questions** | **HTA Domain^a^** | **Issue ID^a^ (Reference)** |
| --- | --- | --- | --- | --- |
| 1 | Low extra costs (data usage &  personal technology) for users and carers | Do/will health patients/carers invest in the personal digital technologies (mobiles/tablets) and data usage fees required to use the DHT? Is it costly/difficult to support? | CUR | A0011/2 (2, 32) |
| 2 | Easy to access and use for everyone | Is the DHT limited in terms of platforms, languages, network connectivity, or users’ digital literacy? | CUR | A0011/2 (2, 32) |
|  |  | Is the DHT designed to minimize the barriers associated with hardware, software, data requirements, and platform services, or the language/location, age, culture, and ability of users? | SAF | DHT05 (21, 36, 57) |
|  |  | Is there evidence DHT is usable and accessible for a diverse range of users, including those with disabilities or limited technical ability? Are there obvious design issues hindering usability, e.g., washable, durable, cause skin allergies? | EFF | D0017 (20, 41, 44, 49, 51, 54, 57, 59) |
|  |  | How does the DHT overcome access barriers, e.g., patients/with a lack of economic resources, poor IT skills/digital health literacy? Is the DHT compatible with common assistive technologies, meet relevant web page or web application standards and available in a wide number of languages and platforms? | ETH | H0012 (21, 55, 63) |
| 3 | Lets the health service know how many patients are using it, so any improvements can be made | Is(will) data on DHT usage (be)collected and easily accessible ongoing to make future investment decisions? | CUR | A0011/2 (2, 32) |
| 4 | Good training and technical support to keep users safe | Is their training on digital skills, personal data handling, digital health literacy, and cyber-safety for all users along with 24-hour technical support? | TEC | B0013/4 (32, 48, 49, 63) |
| 5 | Always records the correct information about patients | How well do the DHT and comparator(s) perform in overcoming technical barriers such as interoperability and data extraction? | TEC | DHT01 (21, 41, 48, 55, 57) |
|  |  | How well is interoperability designed and data quality managed:   - Does the DHT have processes to support the creation and maintenance of accurate healthcare records that can be integrated with multiple information systems using the relevant patient/ provider identifiers and standard terminologies? | SAF | DHT03 (21, 41, 48, 55, 57) |
| 6 | Shows patient information clearly and explains it | How well do the DHT and comparator(s) perform in overcoming technical barriers such as data visualization and feedback? | TEC | DHT01 (54) |
| 7 | The patient can download all their data in a useable format | Is there standardization of access and extraction mechanisms, including the ability for users to extract raw data? | TEC | DHT01 (48, 54) |
| 8 | Low extra costs (equipment, IT, services) for the health service to support it | What investment/tools are required:   - Have device dimensions, battery life and charging methods, calibration requirements, operational system compatibility, connectivity requirements (e.g., wired, Wi-Fi, Bluetooth), data access and storage, data security, technical and data support been considered? | TEC | B0007(20, 35, 41, 49, 54, 57, 63) |
|  |  | Have costs of supporting health care providers in using DHT and costs to use the DHT in the health system (licensing, platforms, hardware, etc.) been considered, e.g.:   - Training, help desks, and change management system - Platform, licensing, attachable hardware, and versions of DHT that would be used in the health system - The need for additional or recurrent purchases, shipping fees, or technical support subscription charges, as well as relevant supply information, such as availability in the target country and minimum order requirements | ECO | E0001/2/9 (20, 29, 54, 63), |
|  |  | Given all the functionalities of DHTs may not be used, and many people may not use the DHT from the outset, are the estimated benefits of the DHT realistic? | ECO | E0005 (32) |
|  |  | Are within-trial collected costs and outcomes externally valid? Are changes in fixed costs for scaling up the DHT known? Is the cost function per patient smooth or stepped? | ECO | DHT20 (20) |
| 9 | Ensures patient information is always kept private and safe from hacking | How well are data security and privacy managed?   - Does it comply with GDPR principles of data minimization/protection by default/design? - Does the DHT comply with data protection legislation/standards - Does it allow users to manage access to their data? - Has the DHT been regularly audited for actual data transmissions to third parties and is the user informed of this risk? - Does the DHT employ authentication, encryption, and threat analysis to avoid unauthorized access to personal data? - Is there safeguarding around peer-to-peer and other communications within DHT platforms? | SAF | DHT02 (2, 21, 31, 41, 48, 51, 55, 57) |
|  |  | - Where are alerts about a patient’s health reported? - Is real-time data securely transmitted? - How does the DHT affect the participant’s safety and welfare? - Have there been any perceived or real privacy breaches, technical problems, unexpected/unintended incidents created by the DHT? | ETH | F0003 (35, 45) |
|  |  | - Does the DHT clearly identify who holds any personal data? - Is the supplier’s cookie policy stated and clear? Is only data necessary for a particular treatment is shared with the doctor and only after explicit consent, which the patient can revoke? - Can patients opt-out if they are not able or unwilling to manage their data? - Does the DHT provider have privacy policies that are easy to understand, uphold users’ rights and choices, and are readily available to users before and while using the DHT, compliant with privacy laws, privacy principles, and best practices? - Are changes to privacy policies communicated to users in a timely way? | ETH | F0101 (21, 31, 41, 48) |
| 10 | There is enough information for users to know how it works and what could go wrong | How transparent are the DHT risks (e.g., data sharing, conflicts of interest) to the user?   - Does the DHT provide users with accurate information on how their data is collected, used, protected, and shared? - Is there clear identification of the DHT’s owners, contact information, funding sources, promotion and sponsorship, and any other possible conflicts of interest? | SAF | DHT04 (21, 35, 36, 41, 48, 55, 57, 63) |
|  |  | Is adequate information disclosed on DHT algorithms to evaluate their risk?   - Has data quality been validated prior to building and employing algorithms? - Are data quality checks built programmatically into artificial intelligence algorithms to avoid harm? - Does the developer/manufacturer clearly state the limitations of the data used, algorithms deployed, especially any learning algorithms, and how outcomes are validated to users? - For learning algorithms, is there adequate disclosure of the characteristics of the training, test, and validation data, the model, and the algorithms to understand how the algorithm controls the clinical decision-making process? | SAF | DHT06 (23,55,56,57) |
|  |  | Are expected direct and data usage costs made clear to the user to improve adherence rates? | SOC | H0203 (21, 32-34) |
| 11 | It is highly reliable and stable | How technically reliable and stable are the DHT and comparator(s):   - Is there evidence of accurate and reliable transmission of unbiased data? - Does the DHT alert the user when working suboptimally or experiencing interference, e.g., low or no network connectivity? - Does it perform well outside the laboratory, and is it validated for use on multiple platforms? - Is it resilient to erroneous data inputs, errors of precision, hardware problems, inappropriate use of devices, changes in other applications, and other interruptions? | SAF | DHT08 (2, 20, 21, 38, 41, 51, 56, 57, 60, 63) |
|  |  | How well are updates/continuity of the DHT managed:   - Is there evidence that platform and operating system updates and patches, service continuity, backup, and recovery mechanisms are well managed? | SAF | DHT08 (21, 63) |
| 12 | Helps health professionals respond quickly when changes in patient care are needed | - Does the DHT allow the user to communicate to the provider critical information about changes in their condition or information on risks of the DHT? - Is there a contact mechanism for technical support with a fixed response time? - Are there processes within the DHT to:   - Correctly identify users?   - Communicate changes to or transfer of a patient’s care? | SAF | DHT09/10/11 (21, 36) |
| 13 | Has additional benefits – patients more confident in their managing condition, less travel and waiting, more connected health team | Have DHT specific outcomes been considered and measured where possible, e.g., improved access to health information and services, reduced waiting time, less burdensome travels, a feeling of security, transfer of skills, better-managed care through self-management and digitally connected healthcare professionals? | ECO | E0005 (20,29,48) |
| 14 | The health advice it provides is always up-to-date and correct | Is there evidence that the health information provided by the DHT is accurate, valid, up to date, sufficiently comprehensive, clear, tailored to the users' diversity, and that there are quality assurance processes in place? | EFF | DHT16 (2, 20, 21, 35, 36, 41, 51, 60, 63) |
| 15 | When trying to change patient habits, it uses best practice and respected methods | Are appropriate and best practice behavior change techniques used in the technology?  Is the targeted behavior change apparent to the user, is the mechanism is credible, are the appropriate supports in place, and it is relevant for the target population? | EFF | DHT17 (2, 35, 39, 41, 43, 44, 47, 51) |
| 16 | Patients and caregivers helped design it and were happy with it | Were patients satisfied with the technology?   - Is there evidence to show relevant stakeholders were involved in the design and satisfied with the DHT? - Is there ongoing data collected on user satisfaction that will be acted upon and available to decision-makers? - Has qualitative data been collected and analyzed to evaluate the mode of action, differences between recipients and sites and identify barriers to uptake or implementation? - Does the DHT create additional burdens on the patient or caregiver, which may affect uptake or adherence? | EFF | D0017 (2, 20, 21, 35, 36, 41, 43, 44, 51,57, 59, 62) |
| 17 | Can be used anywhere | Can the results be transferred to other patient groups/settings/regions?   - Will it work in regions where telecommunication infrastructure is poor, or there is low network connectivity? | EFF | DHT19 (20,35,59) |
|  |  | Is there evidence of the DHT being used in hard-to-reach populations? | ETH | H0012 (2) |
|  |  | - How much does the DHT improve the connectivity between the healthcare team and the patient? - Is access improved for remote patients? | SOC | H0201 (49) |
| 18 | Does not limit the user in their treatment options | Is the user always able to make independent and authentic decisions based on an adequate range of options given by the DHT?  Does the DHT use clear and simple language, providing:   - Concise information how health information was chosen and who is responsible for content? - Controls to prevent behaviour change for purposes other than those stated; e.g. commercial purposes? - Information on potential conflicts of interest (funding, promotion)   Is the DHT designed and used for clearly defined purposes that uphold the health system's social values or society’s values? | ETH | F0004 (30)  F0005 (41)  F0011 (52) |
| 19 | Prevents patients misinterpreting test results or having a false sense of security | Could patients have a false sense of security if their DHT is collecting real-time data and not being contacted by physicians?  Are there harms from the patient having access to the data without someone's assistance to help them interpret what it means?  What will be done with any incidental findings? | ETH | F0003(45) |
| 20 | The new care pathway is mapped out, staff can adapt to it easily, and they have the resources they need | Have the steps in the proposed new care pathway or pathways incorporating the DHT intervention for the relevant population and setting been detailed?  Have infrastructure and service-level changes to existing pathways and associated systems to implement, operate and maintain the new pathway been identified?  What changes are required to staff work methods, staff communication and interactions, electronic communications and information/reporting systems?  How prepared is the health service to make these changes? How does removing the constraints of distance and sharing patient data impact staff work methods and the interactions between medical staff, patients, and their carers?  Have all contextual barriers and enablers to DHT uptake: Infrastructure, clinical endorsement, champions of DHT, supplementary payments, etc been considered? | ORG | G0004 (20)  G0100 (2, 23, 62)  DHT21 (32-34) |
| 21 | Relevant health professionals have been involved in the design and they support its use | Does the DHT have credibility with health care professionals? Is there published or publicly available evidence documenting the relevant health care experts' role in the design, development, testing, or sign-off of the DHT? Enablers: Are there are champions of DHT within the health service? | ORG | G0010 (2, 21, 60)  DHT21 (32-34) |
| 22 | It is clear who is legally responsible for what and who owns the data | Are parties responsible for medical advice, responsible for monitoring and reviewing patient data, and that own the data related to the DHT, clearly defined?  Are litigation risks to the healthcare practitioners, and how insurance(s) (i.e., professional indemnity, life, health, income) and professional registrations could be affected through use or recommendation of the DHT, clearly defined? | LEG | DHT22 (63) |
| 23 | With patient consent, their data can be easily linked to existing medical records for clinician review | Does the DHT have processes to support the creation and maintenance of accurate healthcare records that can be integrated with multiple information systems using the relevant patient/ provider identifiers and standard terminologies? | TEC | DHT01 (21, 41, 48, 55, 57) |
|  |  | How well is interoperability designed? | SAF | DHT03 (21, 41, 48, 55, 57) |
| 24 | At is at least as effective as usual (face to face) care | Has effectiveness been demonstrated:   - Are accepted methods used to overcome common methodological problems in RCTs for DHTs, e.g., achieving blinding, biases from informed consent? - Is it clear whether the DHT was changed (bug fixes, content) during the trial? - Was digital literacy an implicit eligibility criterion? - Was the comparator group restricted in the DHT to which they had access? - Have DHT-specific and validated outcome measures been collected: i.e., the intensity of use (dose, exposure), online adherence, engagement? - Has data collection embedded in the DHT created systematic bias? - Is reporting of the RCT in accordance with CONSORT E-HEALTH? | EFF | DHT12 (22, 23, 35, 39)  DHT13 (44, 47)  DHT14 (2, 35, 50, 53) |
|  |  | Are the results external valid/generalizable:   - Have the actions taken to enhance the trial’s internal validity, such as participant identity validation and obtaining off-line contact details to promote good follow-up rates, skewed participant populations, and jeopardized external validity? - Are the results generalizable to the general internet population, to the general patient population, or other organizations? | EFF | DHT18 (44, 47)  DHT19 (20, 59) |

^a^ From EUNetHTA’s HTA Core Model version 3.0 or DHT specific issue identifier ^2^

References for Supplementary Table 1

1. Australian Institute of Health and Welfare. Chronic disease overview. 2020 [cited 2021 Feb 10]; Available from: https://www.aihw.gov.au/reports-data/health-conditions-disability-deaths/chronic-disease/overview.

2. National Institute for Health and Care Excellence (UK). Evidence standards framework for digital health technologies. London (UK): NICE (UK); 2021

3. Medical Device Coordination Group. Guidance on qualification and classification of software in regulation (EU) 2017/745 – MDR and Regulation (EU) 2017/746 – IVDR. European Commission; 2019.

4. O'Rourke B, Oortwijn W, Schuller T. The new definition of health technology assessment: A milestone in international collaboration. *Int J Technol Assess Health Care*. 2020;36(3):187-90.

5. Australian Government Department of Health and Ageing. Review of health technology assessment in Australia. Canberra (AU): Commonwealth of Australia; 2009.

6. Regulation (EU) 2017/745 of the European Parliament and of the Council (2017) *Official Journal* L117, p. 1-175.

7. Regulation (EU) 2016/679 of the European Parliament and of the Council (2016) *Official Journal* L119, p. 1-88.

8. Moshi MR, Tooher R, Merlin T. Suitability of current evaluation frameworks for use in the health technology assessment of mobile medical applications: A systematic review. *Int J Technol Assess Health Care*. 2018 Jan;34(5):464-75.

9. Iribarren SJ, Cato K, Falzon L, Stone PW. What is the economic evidence for mhealth? A systematic review of economic evaluations of mhealth solutions. *PLoS One*. 2017;12(2):e0170581.

10. Kidholm K, Kristensen MBD. A scoping review of economic evaluations alongside randomized controlled trials of home monitoring in chronic disease management. *Applied Health Economics and Health Policy*. 2018;16(2):167-76.

11. Vukovic V, Favaretti C, Ricciardi W, de Waure C. Health technology assessment evidence on e-health/m-health technologies: Evaluating the transparency and thoroughness. *Int J Technol Assess Health Care*. 2018 Jan;34(1):87-96.

12. Moher D, Liberati A, Tetzlaff J, Altman DG. Preferred reporting items for systematic reviews and meta-analyses: The PRISMA statement. *PLoS Med*. 2009;6(7):e1000097.

13. Grey matters: A practical tool for searching health-related grey literature. [Internet] Ottawa (CA): CADTH; 2018 [updated 2019 Apr; cited 2020 Apr 4]; Available from: https://www.cadth.ca/resources/finding-evidence.

14. Hailey D, Ohinmaa A, Roine R. Study quality and evidence of benefit in recent assessments of telemedicine. London (UK): SAGE Publications; 2004. p. 318-24.

15. Gagnon M-P, Scott R. Striving for evidence in e-health evaluation: Lessons from health technology assessment. *J Telemed Telecare*. 2005;11:S34-6.

16. Reardon T. Research findings and strategies for assessing telemedicine costs. *Telemedicine and e-Health*. 2005;11(3):348-69.

17. Shiell A, Hawe P, Gold L. Complex interventions or complex systems? Implications for health economic evaluation. *BMJ*. 2008;336(7656):1281-3.

18. Dávalos ME, French MT, Burdick AE, Simmons SC. Economic evaluation of telemedicine: Review of the literature and research guidelines for benefit-cost analysis. *Telemedicine and e-Health*. 2009;15(10):933-48.

19. EUnetHTA Joint Action 2, Work Package 8. HTA Core Model® version 3.0. [Pdf]; 2016. Available from: www.htacoremodel.info/BrowseModel.aspx.

20. Kidholm K, Ekeland AG, Jensen LK, Rasmussen J, Pedersen CD, Bowes A, et al. A model for assessment of telemedicine applications: MAST. *Int J Technol Assess Health Care*. 2012;28(1):44.

21. Australian Commission on Safety and Quality in Health Care. National safety and quality digital mental health standards - consultation draft. Sydney (AU): The Commission; 2020.

22. Haute Autorité de Santé [French National Authority for Health]. Methodological choices for the clinical development of medical devices. Paris (FR): The Authority; 2013.

23. Haute Autorité de Santé [French National Authority for Health]. Guide to the specific features of clinical evaluation of a connected medical device (cmd) in view of its application for reimbursement. Paris (FR): The Authority; 2019.

24. Mookherji S, Mehl G, Kaonga N, Mechael P. Unmet need: Improving mhealth evaluation rigor to build the evidence base. *Journal of Health Communication*. 2015;20(10):1224-9.

25. Philpott D, Guergachi A, Keshavjee K. Design and validation of a platform to evaluate mhealth apps. *Stud Health Technol Inform*. 2017 April 2017;235:3-7.

26. Wyatt JC. How can clinicians, specialty societies and others evaluate and improve the quality of apps for patient use? *BMC Med*. 2018;16(1):225.

27. McNamee P, Murray E, Kelly MP, Bojke L, Chilcott J, Fischer A, et al. Designing and undertaking a health economics study of digital health interventions. *Am J Prev Med*. 2016;51(5):852-60.

28. Rickles D, Hawe P, Shiell A. A simple guide to chaos and complexity. *J Epidemiol Community Health*. 2007;61(11):933-7.

29. Bergmo TS. How to measure costs and benefits of ehealth interventions: An overview of methods and frameworks. *J Med Internet Res*. 2015;17(11):e254.

30. Sax M, Helberger N, Bol N. Health as a means towards profitable ends: Mhealth apps, user autonomy, and unfair commercial practices. *Journal of Consumer Policy*. 2018;41(2):103-34.

31. Huckvale K, Torous J, Larsen ME. Assessment of the data sharing and privacy practices of smartphone apps for depression and smoking cessation. *JAMA Network Open*. 2019;2(4):e192542.

32. Drury P, Roth S, Jones T, Stahl M, Medeiros D. Guidance for investing in digital health. Manila (PH): Asian Development Bank (ADB); 2018.

33. Lennon MR, Bouamrane MM, Devlin AM, O'Connor S, O'Donnell C, Chetty U, et al. Readiness for delivering digital health at scale: Lessons from a longitudinal qualitative evaluation of a national digital health innovation program in the United Kingdom. *J Med Internet Res*. 2017 02 16;19(2):e42.

34. Rojahn K, Laplante S, Sloand J, Main C, Ibrahim A, Wild J, et al. Remote monitoring of chronic diseases: A landscape assessment of policies in four European countries. *PLoS One*. 2016;11(5):e0155738.

35. Eysenbach G. Consort-ehealth: Improving and standardizing evaluation reports of web-based and mobile health interventions. *J Med Internet Res*. 2011;13(4):e126.

36. Andalusian Health Quality Agency (ES). Complete list of recommendations on design, use and assessment of health apps. [Internet] Seville (ES): 2012 [cited 2020 Aug 16]; Available from: www.calidadappsalud.com/en/listado-completo-recomendaciones-app-salud/.

37. Khoja S, Durrani H, Scott RE, Sajwani A, Piryani U. Conceptual framework for development of comprehensive e-health evaluation tool. *Telemedicine and e-Health*. 2013;19(1):48-53.

38. Lewis TL, Wyatt JC. Mhealth and mobile medical apps: A framework to assess risk and promote safer use. *J Med Internet Res*. 2014 Sep 15;16(9):e210.

39. Mohr DC, Schueller SM, Riley WT, Brown CH, Cuijpers P, Duan N, et al. Trials of intervention principles: Evaluation methods for evolving behavioral intervention technologies. *J Med Internet Res*. 2015;17(7):e166.

40. Steventon A, Grieve R, Bardsley M. An approach to assess generalizability in comparative effectiveness research: A case study of the whole systems demonstrator cluster randomized trial comparing telehealth with usual care for patients with chronic health conditions. *Med Decis Making*. 2015 11;35(8):1023-36.

41. Ruck A, Wagner Bondorf S, Lowe C (Consard Limited). Second draft of guidelines, EU guidelines on assessment of the reliability of mobile health applications. European Commission, Directorate-General of Communications Networks, Content & Technology; 2016.

42. Gorski I, Bram JT, Sutermaster S, Eckman M, Mehta K. Value propositions of mhealth projects. *J Med Eng Technol*. 2016 16 Nov;40(7-8):400-21.

43. McMillan B, Hickey E, Patel MG, Mitchell C. Quality assessment of a sample of mobile app-based health behavior change interventions using a tool based on the national institute of health and care excellence behavior change guidance. *Patient Educ Couns*. 2016;99(3):429-35.

44. Murray E, Hekler EB, Andersson G, Collins LM, Doherty A, Hollis C, et al. Evaluating digital health interventions: Key questions and approaches. *Am J Prev Med*. 2016;51(5):843-51.

45. IRBs could address ethical issues related to tracking devices: Mobile devices raise new concerns. *IRB Advisor*. 2017 Nov;17(11):8-9.

46. Maar MA, Yeates K, Perkins N, Boesch L, Hua-Stewart D, Liu P, et al. A framework for the study of complex mhealth interventions in diverse cultural settings. *JMIR MHealth and UHealth*. 2017 Apr 20;5(4):e47.

47. Michie S, Yardley L, West R, Patrick K, Greaves F. Developing and evaluating digital interventions to promote behavior change in health and health care: Recommendations resulting from an international workshop. *J Med Internet Res*. 2017;19(6):e232.

48. European Commission. Synopsis report, Consultation: Transformation health and care in the digital single market. Luxembourg: The Commission; 2018.

49. Hogaboam LS. Assessment of technology adoption potential of medical devices: Case of wearable sensor products for pervasive care in neurosurgery and orthopedics [Ph.D.]. Ann Arbor: Portland State University; 2018.

50. Jurkeviciute M. Planning of a holistic summative ehealth evaluation: The interplay between standards and reality [Licentiate]. Ann Arbor: Chalmers Tekniska Hogskola (Sweden); 2018.

51. Nielsen S, Rimpiläinen S. Report on international practice on digital apps. Glasgow (UK): Digital Health and Care Institute; 2018.

52. Academy of Medical Sciences (UK). Our data-driven future in healthcare: People and partnerships at the heart of health related technologies. London (UK): The Academy; 2018.

53. Beintner I, Vollert B, Zarski AC, Bolinski F, Musiat P, Gorlich D, et al. Adherence reporting in randomized controlled trials examining manualized multisession online interventions: Systematic review of practices and proposal for reporting standards. *J Med Internet Res*. 2019 08 15;21(8):e14181.

54. Caulfield B, Reginatto B, Slevin P. Not all sensors are created equal: A framework for evaluating human performance measurement technologies. *NPJ Digital Medicine*. 2019;2(1).

55. Department of Health & Social Care (UK). Code of conduct for data-driven health and care technology. [Internet] London (UK): The Department; 2019 [updated 2019 Jul 18; cited 2020 Aug 18]; Available from: https://www.gov.uk/government/publications/code-of-conduct-for-data-driven-health-and-care-technology/initial-code-of-conduct-for-data-driven-health-and-care-technology.

56. Haute Autorité de Santé [French National Authority for Health]. Public consultation on the draft analysis grid intended for use by CNEDiMTS to contribute to its evaluation of medical devices embedding decision systems based on automatic learning processes ("artificial intelligence"). Paris (FR): The Authority; 2019.

57. NHS Digital (UK). How we assess health apps and digital tools. [Internet] London (UK): NHS Digital; 2019 [updated 2019 May 17; cited 2020 Apr 13]; Available from: https://digital.nhs.uk/services/nhs-apps-library/guidance-for-health-app-developers-commissioners-and-assessors/how-we-assess-health-apps-and-digital-tools.

58. Rajan B, Tezcan T, Seidmann A. Service systems with heterogeneous customers: Investigating the effect of telemedicine on chronic care. *Management Science*. 2019;65(3):1236-67.

59. Dick S, O'Connor Y, Thompson MJ, O'Donoghue J, Hardy V, Wu TJ, et al. Considerations for improved mobile health evaluation: Retrospective qualitative investigation. *JMIR MHealth and UHealth*. 2020 Jan 22;8(1):e12424.

60. Federal Ministry of Health (DE), Regulation on the procedure and requirements for testing the eligibility for reimbursement of digital health applications in the statutory public health insurance (Digital Health Applications Ordinance - DiGAV) (Draft bill), [Bonn (DE)]: The Ministry; 2020.

61. Health Information and Quality Authority (IE). International review of consent models for the collection, use and sharing of health information. Cork (IE): The Authority; 2020.

62. Medical Services Advisory Committee (AU). Draft guidelines for preparing assessment reports for the medical services advisory committee. Canberra (AU): The Committee; 2020.

63. Moshi MR, Tooher R, Merlin T. Development of a health technology assessment module for evaluating mobile medical applications. *Int J Technol Assess Health Care*. 2020;36(3):252-61.

64. World Health Organization. Who guideline: Recommendations on digital interventions for health system strengthening. Geneva (CH): The Organization; 2019.

Supplementary Table 2: Relative preferences of attributes and class allocation model for three latent class model

| **Relative preferences** | |  | **Latent Class 1** | | | |  | **Latent Class 2** | | | |  | **Latent Class 3** | | | |
| --- | --- | --- | --- | --- | --- | --- | --- | --- | --- | --- | --- | --- | --- | --- | --- | --- |
| **HTA Domain*** | **Attributes** |  | ***β*** | **(95% CI)** | **Preference Score** | ***p*** |  | ***β*** | **(95% CI)** | **Preference Score** | ***p*** |  | ***β*** | **(95% CI)** | **Preference Score** | ***p*** |
| SAF | Helps health professionals respond quickly when changes in patient care are needed |  | 0.41 | (0.12, 0.69) | 19.2 | 0.006 |  | 2.49 | (2.03, 2.96) | 77.4 | <0.001 |  | 3.31 | (2.61, 4.01) | 100.0 | <0.001 |
| TEC/SAF | Always records the correct information about patients |  | 0.70 | 0.42, 0.99) | 27.5 | <0.001 |  | 2.12 | (1.76, 2.48) | 67.0 | <0.001 |  | 2.81 | (2.32, 3.31) | 86.3 | <0.001 |
| EFF | The health advice it provides is always up-to-date and correct |  | 0.24 | (-0.03, 0.52) | 14.7 | 0.086 |  | 1.91 | (1.56, 2.26) | 61.1 | <0.001 |  | 2.69 | (2.24, 3.15) | 82.9 | <0.001 |
| SAF | It is highly reliable and stable |  | 0.72 | (0.41, 1.02) | 27.8 | <0.001 |  | 1.87 | (1.55, 2.20) | 60.1 | <0.001 |  | 2.61 | (2.22, 3.01) | 80.7 | <0.001 |
| EFF | It is at least as effective as usual (face-to-face) care |  | -0.27 | (-0.52, -0.03) | 0.3 | 0.030 |  | 0.47 | (-0.06, 0.99) | 20.9 | 0.085 |  | 2.14 | (1.40, 2.88) | 67.5 | <0.001 |
| EFF/ECO | Has additional benefits - patients more confident in their managing condition, less travel and waiting, more connected health team |  | 0.49 | (0.21, 0.76) | 21.4 | <0.001 |  | 1.41 | (0.87, 1.96) | 47.2 | <0.001 |  | 2.12 | (1.38, 2.86) | 67.0 | <0.001 |
| ETH | Does not limit the user in their treatment options |  | -0.13 | (-0.39, 0.13) | 4.3 | 0.333 |  | 0.68 | (0.32, 1.03) | 26.7 | <0.001 |  | 1.80 | (1.28, 2.31) | 58.0 | <0.001 |
| ETH | Prevents patients misinterpreting test results or having a false sense of security |  | 0.01 | (-0.26, 0.28) | 8.1 | 0.957 |  | 1.35 | (1.02, 1.68) | 45.6 | <0.001 |  | 1.78 | (1.37, 2.19) | 57.6 | <0.001 |
| TEC | Good training and technical support to keep users safe |  | 0.46 | (0.21, 0.71) | 20.7 | <0.001 |  | 1.26 | (0.94, 1.58) | 43.0 | <0.001 |  | 1.59 | (1.20, 1.99) | 52.3 | <0.001 |
| TEC/SAF | With patient consent, their data can be easily linked to existing medical records for clinician review |  | -0.11 | (-0.36, 0.13) | 4.8 | 0.371 |  | 1.61 | (1.13, 2.09) | 52.7 | <0.001 |  | 1.59 | (1.05, 2.13) | 52.3 | <0.001 |
| TEC | Shows patient information clearly and explains it |  | 0.54 | (0.30, 0.79) | 23.1 | <0.001 |  | 1.36 | (0.97, 1.74) | 45.7 | <0.001 |  | 1.55 | (1.11, 1.99) | 51.2 | <0.001 |
| SAF | Ensures patient information is always kept private and safe from hacking |  | 0.55 | (0.24, 0.86) | 23.3 | <0.001 |  | 2.94 | (2.44, 3.44) | 89.7 | <0.001 |  | 1.45 | (0.97, 1.94) | 48.3 | <0.001 |
| CUR/SAF/ EFF/ETH | Easy to access and use for everyone |  | 0.68 | (0.43, 0.94) | 26.9 | <0.001 |  | 0.87 | (0.46, 1.28) | 32.2 | <0.001 |  | 1.29 | (0.79, 1.78) | 43.8 | <0.001 |
| ORG | The new care pathway is mapped out, staff can adapt to it easily, and they have the resources they need |  | -0.02 | (-0.27, 0.22) | 7.2 | 0.845 |  | 0.97 | (0.58, 1.37) | 35.0 | <0.001 |  | 1.14 | (0.62, 1.66) | 39.6 | <0.001 |
| SAF | There is enough information for users to know how it works and what could go wrong |  | 0.29 | (0.05, 0.53) | 16.0 | 0.018 |  | 0.59 | (0.26, 0.92) | 24.3 | <0.001 |  | 1.05 | (0.60, 1.49) | 37.0 | <0.001 |
| ORG | Relevant health professionals have been involved in the design and they support its use |  | 0.08 | (-0.15, 0.30) | 10.1 | 0.494 |  | 0.83 | (0.53, 1.13) | 31.1 | <0.001 |  | 0.81 | (0.46, 1.16) | 30.6 | <0.001 |
| EFF | When trying to change patient habits, it uses best practice and respected methods |  | -0.05 | (-0.28, 0.18) | 6.5 | 0.665 |  | 0.75 | (0.41, 1.09) | 28.8 | <0.001 |  | 0.81 | (0.39, 1.23) | 30.4 | <0.001 |
| EFF/ETH/  SOC | Can be used anywhere |  | 0.07 | (-0.19, 0.32) | 9.7 | 0.610 |  | 0.10 | (-0.29, 0.48) | 10.6 | 0.615 |  | 0.62 | (0.15, 1.09) | 25.2 | 0.009 |
| CUR | Low extra costs (data usage & personal technology) for users and carers |  | 0.83 | (0.55, 1.11) | 31.1 | <0.001 |  | 0.17 | (-0.18, 0.53) | 12.7 | 0.333 |  | 0.58 | (0.12, 1.04) | 24.0 | 0.013 |
| TEC/ECO | Low extra costs (equipment, IT, services) for the health service to support it |  | 0.48 | (0.23, 0.73) | 21.2 | <0.001 |  | -0.17 | (-0.52, 0.18) | 3.2 | 0.345 |  | 0.54 | (0.03, 1.04) | 22.9 | 0.036 |
| TEC | The patient can download all their data in a useable format |  | 0.01 | (-0.23, 0.26) | 8.3 | 0.911 |  | 0.54 | (0.18, 0.89) | 22.9 | 0.003 |  | 0.37 | (-0.06, 0.80) | 18.2 | 0.090 |
| CUR | Lets the health service know how many patients are using it, so any improvements can be made |  | 0.31 | (0.07, 0.56) | 16.6 | 0.012 |  | -0.09 | (-0.48, 0.30) | 5.4 | 0.647 |  | 0.24 | (-0.21, 0.69) | 14.6 | 0.291 |
| LEG | It is clear who is legally responsible for what and who owns the data |  | -0.28 | (-0.52, -0.04) | 0.0 | 0.020 |  | 0.85 | (0.44, 1.26) | 31.5 | <0.001 |  | -0.26 | (-0.76, 0.25) | 0.7 | 0.317 |
| EFF | Patients and caregivers helped design it and were happy with it |  | Reference | | | |  | Reference | | | |  | Reference | | | |

| **Class allocation model** |  | **Latent Class 1** | | | |  | **Latent Class 2** | | | |  | **Latent Class 3** | | | |
| --- | --- | --- | --- | --- | --- | --- | --- | --- | --- | --- | --- | --- | --- | --- | --- |
| **Variable** |  | **OR** | **(95% CI)** |  | ***p*** |  | **OR** | **(95% CI)** |  | ***p*** |  | **OR** | **(95% CI)** |  | ***p*** |
| **Respondent type** |  |  |  |  |  |  |  |  |  |  |  |  |  |  |  |
| Patient or carer (reference Community member) |  | 1.32 | (0.86, 2.05) |  | 0.208 |  | 0.63 | (0.41, 0.98) |  | 0.041 |  | Reference class | | | |
| Health professional (reference Community member) |  | 0.80 | (0.33, 1.94) |  | 0.624 |  | 1.58 | (0.56, 4.49) |  | 0.392 |  |  |  |  |  |
| **Gender** |  |  |  |  |  |  |  |  |  |  |  |  |  |  |  |
| Female (reference Male) |  | 0.53 | (0.33, 0.85) |  | 0.008 |  | 1.47 | (0.88, 2.45) |  | 0.141 |  | Reference class | | | |
| **Age group** |  |  |  |  |  |  |  |  |  |  |  |  |  |  |  |
| 40 to 69 yrs (reference 18 to 39 yrs) |  | 0.44 | (0.25, 0.75) |  | 0.003 |  | 1.27 | (0.67, 2.39) |  | 0.467 |  |  |  |  |  |
| 70yrs and over (reference 18 to 39 yrs) |  | 0.16 | (0.05, 0.46) |  | <0.001 |  | 1.07 | (0.45, 2.55) |  | 0.878 |  |  |  |  |  |
| **Country of residence** |  |  |  |  |  |  |  |  |  |  |  |  |  |  |  |
| Canada (reference Australia) |  | 0.98 | (0.53, 1.81) |  | 0.943 |  | 1.75 | (0.89, 3.43) |  | 0.104 |  | Reference class | | | |
| NZ (reference Australia) |  | 0.52 | (0.27, 1.00) |  | 0.051 |  | 1.07 | (0.62, 1.85) |  | 0.803 |  |  |  |  |  |
| UK and other countries (reference Australia) |  | 0.89 | (0.51, 1.57) |  | 0.694 |  | 1.23 | (0.68, 2.22) |  | 0.493 |  |  |  |  |  |
| **Speak a second language at home** |  |  |  |  |  |  |  |  |  |  |  |  |  |  |  |
| No (reference Yes) |  | 0.55 | (0.31, 1.00) |  | 0.051 |  | 0.75 | (0.38, 1.48) |  | 0.411 |  | Reference class | | | |
| **Employment status** |  |  |  |  |  |  |  |  |  |  |  |  |  |  |  |
| Part-time/Casual/Student (reference fulltime) |  | 0.49 | (0.26, 0.93) |  | 0.030 |  | 0.78 | (0.41, 1.46) |  | 0.431 |  | Reference class | | | |
| Not employed/unable to work (reference fulltime) |  | 0.81 | (0.41, 1.60) |  | 0.542 |  | 0.85 | (0.38, 1.88) |  | 0.686 |  |  |  |  |  |
| Retired (reference fulltime) |  | 0.33 | (0.15, 0.74) |  | 0.007 |  | 0.82 | (0.42, 1.61) |  | 0.563 |  |  |  |  |  |
| **How often do you need someone to help you when using your computer, mobile phone, tablet, or smart watch?** | | | | | | | | | |  |  |  |  |  |  |
| Sometimes/Often/Always (reference None/Rarely) |  | 5.30 | (2.98, 9.42) |  | <0.001 |  | 1.48 | (0.80, 2.73) |  | 0.213 |  | Reference class | | | |
| *Average class probability* |  | *0.30* | | | |  | *0.38* | | | | | *0.32* | | | |

Model Fit: AIC = 48254.47 (lowest out of all models with covariates added to class membership model), Respondents: n = 1,251, Pseudo r2 = 0.094, Proportion of participants classified in each latent class with a posterior probability above 75%: Class 1: 80%, Class 2: 66%, Class 3: 67%. Means of the posterior probabilities of belonging to the latent class among the subjects classified a posteriori in each latent class: Class 1: 88%, Class 2: 81%, Class 3: 81%. β = Regression model coefficient estimates

*HTA Domain = Health Technology Assessment (HTA) Domains of the EUNetHTA HTA Core Model version 3.0^1^:

CUR: Describes the new technology’s target population, target condition and current management, current and expected utilization, and regulatory status

TEC: Describes the new technology’s features in enough detail to differentiate it from comparators, and the investments, tools, and training required to use it

SAF: Identifies unwanted or harmful effects of the new technology important to patients or the decisions of health care providers and policymakers

EFF: Provides evidence of comparative effectiveness of the new technology in producing health benefits in the relevant health care setting

ECO: Provides information on the new technology’s costs, health-related outcomes, and economic efficiency to inform value for money judgments

ETH: Considers potential harms to autonomy, respect for persons, justice, and equity from the use of the new technology or from performing the HTA

ORG: Identifies resources to mobilized or organized to implement the new technology and the consequences (Intra/inter-organizational and health system)

SOC: Considers issues related to the new technology relevant to patients, carers, and social groups

LEG: Identifies rules and regulations protecting patient’s rights and societal interests for consideration when evaluating the new technology


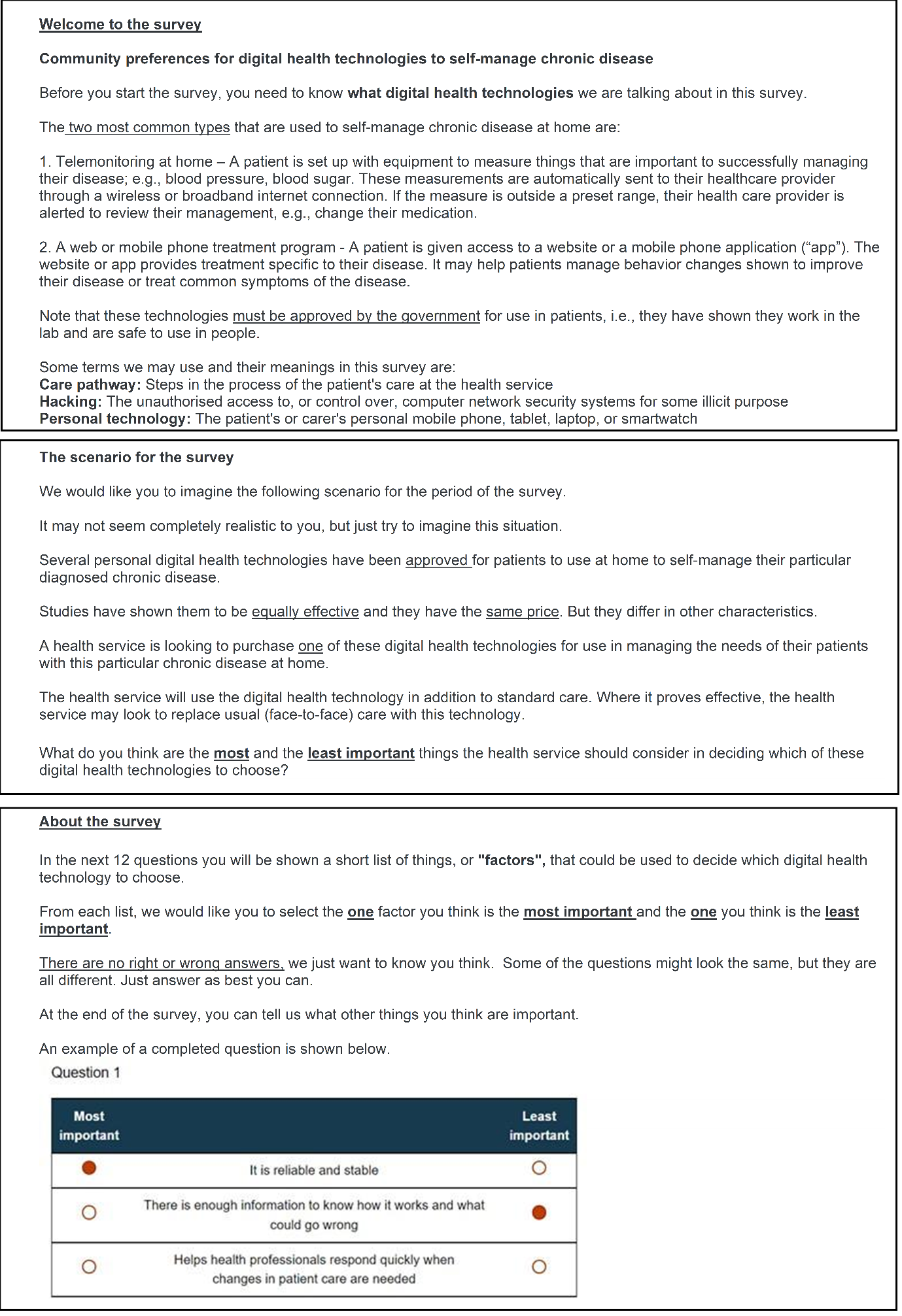


Supplementary Figure 1: Survey preamble with an example of a best-worst choice set


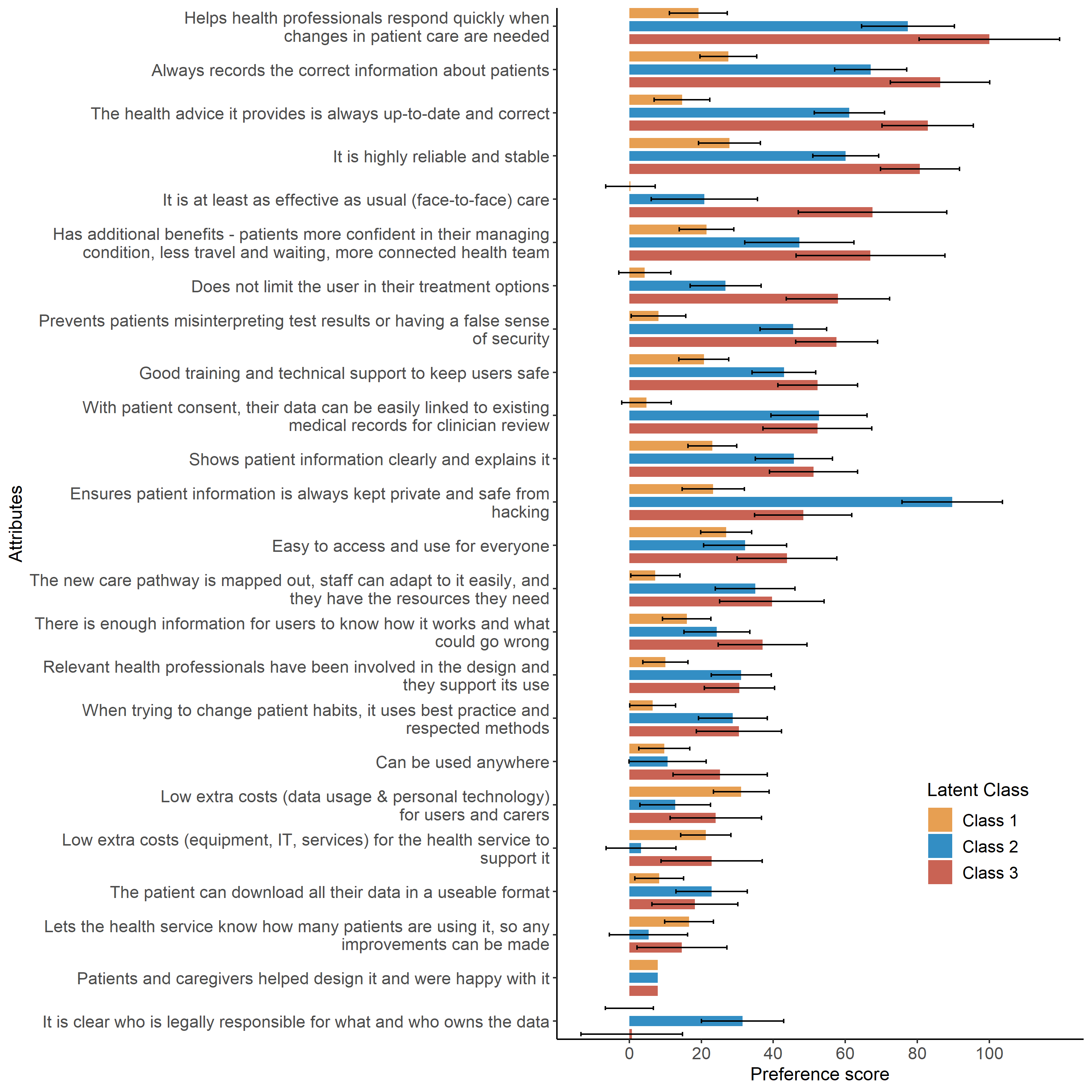


Supplementary Figure 2: Relative preferences for DHT attributes from the sequential best-worst latent class multinomial model
